# Trastuzumab Deruxtecan Significantly Reduces Human Epidermal Growth Factor Receptor 2-Positive Pancreatic Metastasis from Advanced Breast Cancer: A Case Report

**DOI:** 10.70352/scrj.cr.25-0304

**Published:** 2025-10-08

**Authors:** Rino Tanida, Takashige Tanaka, Mai Hamada-Nishimoto, Noriko Senda, Yookija Kang, Toshiki Shiozaki, Hirotake Fujii, Takaki Sakurai, Shigeru Tsuyuki

**Affiliations:** 1 Department of Breast Surgery, Osaka Red Cross Hospital, Osaka, Osaka, Japan; 2 Department of Diagnostic Radiology, Osaka Red Cross Hospital, Osaka, Osaka, Japan; 3 Department of Diagnostic Pathology, Osaka Red Cross Hospital, Osaka, Osaka, Japan

**Keywords:** advanced breast cancer, pancreatic metastasis, trastuzumab deruxtecan

## Abstract

**INTRODUCTION:**

Pancreatic metastases from breast cancer are rare, with only 61 reported cases. They are often difficult to differentiate from primary pancreatic cancer based on clinical presentation and imaging. The median overall survival after the diagnosis of pancreatic metastases has been reported to be short (17 months).

**CASE PRESENTATION:**

A 50-year-old woman was diagnosed with human epidermal growth factor receptor 2 (HER2)-positive advanced right breast cancer, with right axillary lymph node and multiple bone metastases. After palliative irradiation for thoracolumbar metastases, the patient received pertuzumab with trastuzumab plus docetaxel as 1st-line treatment, with a partial response for 9 months. Second-line trastuzumab emtansine maintained stable disease for 14 months (25 months from the initial diagnosis). A follow-up CT scan revealed a pancreatic tumor measuring 48 × 28 mm, which was diagnosed as a metastasis from breast cancer (estrogen receptor: 0%; progesterone receptor: 0%; HER2: 3+; Ki67: 60%) by endoscopic ultrasonography-guided fine-needle biopsy (FNB). MRI revealed a mass in the pancreatic head. Three brain metastases were also detected using head MRI. After stereotactic irradiation of the cerebellar metastases, trastuzumab deruxtecan (T-DXd) was started as 3rd-line treatment, and the pancreatic metastasis was markedly reduced, and a complete response (CR) was achieved, which was effective for 14 months. No severe adverse events were observed during the T-DXd treatment. Capecitabine and lapatinib were administered as 4th-line treatment owing to new pleural dissemination. The patient died of worsening cancerous lymphangitis of the lungs 23 months after the diagnosis of pancreatic metastasis.

**CONCLUSIONS:**

Our patient with HER2-positive pancreatic metastasis achieved CR with marked shrinkage using T-DXd and a progression-free survival of 14 months. FNB should be performed to diagnose possible pancreatic metastases if a pancreatic tumor is detected during breast cancer treatment.

## Abbreviations

CR
: complete response
ER
: estrogen receptor
FNA
: fine-needle aspiration
FNB
: fine-needle biopsy
HER2
: human epidermal growth factor receptor 2
IBC-NST
: invasive breast cancer of no special type
LN
: lymph node
MBC
: metastatic breast cancer
OS
: overall survival
PFS
: progression-free survival
PgR
: progesterone receptor
SD
: stable disease
T-DM1
: trastuzumab emtansine
T-DXd
: trastuzumab deruxtecan

## INTRODUCTION

Pancreatic metastases from primary malignant tumors are rare, constituting approximately 2% of neoplasms that affect the pancreas. The most common primary tumor locations are the kidneys, lungs, skin, colorectum, and breast.^1–3)^ Breast cancer often metastasizes to the bones, lungs, and liver^4,5)^; however, metastases to the pancreas are rare. The incidence of pancreatic metastases from breast cancer varies in clinical settings depending on reports of surgical and fine-needle aspiration (FNA) cases, ranging from 2.5% to 7%.^1,3)^ However, autopsy studies have reported a higher incidence rate of 11%–17%.^6)^

Clinical findings and imaging of pancreatic metastases are often difficult to differentiate from primary pancreatic cancer because of their similarities.^7)^

The development of anti-human epidermal growth factor receptor 2 (HER2) therapy has been remarkable and has significantly improved the prognosis of metastatic breast cancer (MBC). Trastuzumab deruxtecan (T-DXd) is an antibody–drug conjugate composed of an anti-HER2 antibody, a cleavable tetrapeptide-based linker, and a cytotoxic topoisomerase I inhibitor. In the DESTINY-Breast03 trial, T-DXd significantly prolonged both progression-free survival (PFS) and overall survival (OS) compared to trastuzumab emtansine (T-DM1) in HER2-positive MBC with 70% visceral metastases.^8,9)^

Herein, we report a case of pancreatic metastasis from HER2-positive advanced breast cancer in which T-DXd was effective in achieving a complete response (CR).

## CASE PRESENTATION

**Fig. 1:**
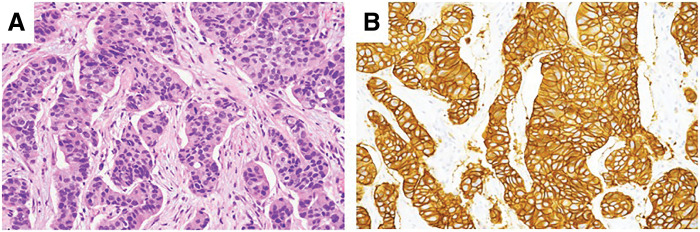
(**A**) Core needle biopsy of the breast showing invasive breast carcinoma of no special type. H&E staining. (**B**) HER2-positive (score 3+) staining (magnification, ×20). H&E, hematoxylin and eosin; HER2, human epidermal growth factor receptor 2

Our patient was a 50-year-old woman who was diagnosed with clinical T4bN1M1 (Stage 4) breast cancer with multiple bone and right axillary lymph node (LN) metastases. Histopathological evaluation of a core needle biopsy revealed that the tumor was an invasive breast carcinoma of no special type (IBC-NST), estrogen receptor (ER)-negative, progesterone receptor (PgR)-negative, HER2: 3+, and Ki67: 60% (**Fig. 1**). After palliative irradiation (40 Gy) for the thoracolumbar metastases, the patient received pertuzumab with trastuzumab plus docetaxel as the 1st-line treatment, resulting in a partial response. Nine months later, the primary tumor and axillary LNs had regrown.

**Fig. 2:**
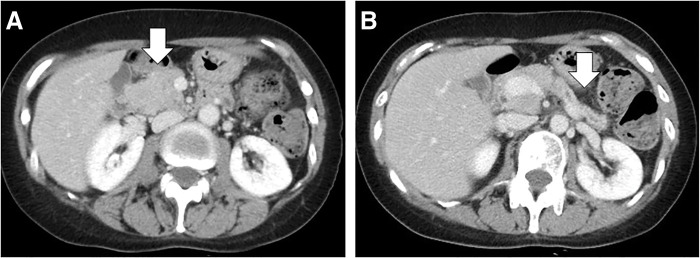
CT images revealing (**A**) a low-density mass measuring 42 × 30 mm in the pancreatic head (arrow) and (**B**) dilatation of the main pancreatic duct (arrow).

Secondary treatment with T-DM1 maintained a stable disease (SD) state. Fourteen months after treatment (25 months after the initial diagnosis), follow-up CT revealed a low-density mass measuring 42 × 30 mm in the pancreatic head with dilatation of the main pancreatic duct. The primary tumor and the metastases to the right axillary LN and bone remained in an SD state (**Fig. 2**).

**Fig. 3:**
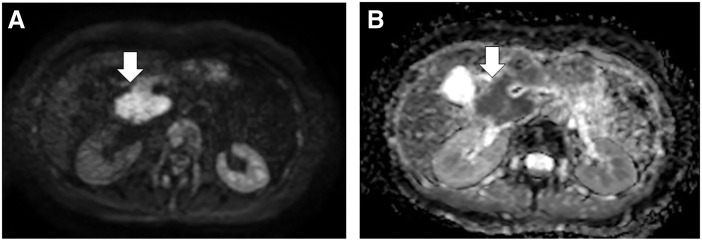
On MRI, (**A**) DWI shows a high-signal mass measuring 48 × 28 mm in the pancreatic head (arrow), and (**B**) the ADC map shows a low-signal mass with clear margins (arrow). ADC, apparent diffusion coefficient; DWI, diffusion-weighted imaging

On MRI, diffusion-weighted imaging showed a high-signal mass measuring 48 × 28 mm in the pancreatic head, and the apparent diffusion coefficient map showed a low-signal mass with clear margins (**Fig. 3**).

**Fig. 4:**
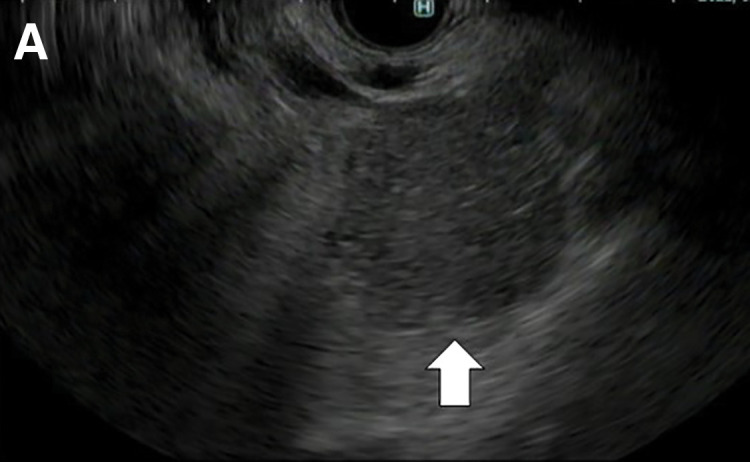
EUS shows a 30-mm hypoechoic mass with irregular margins in the pancreatic head (arrow). EUS, endoscopic ultrasonography

**Fig. 5:**
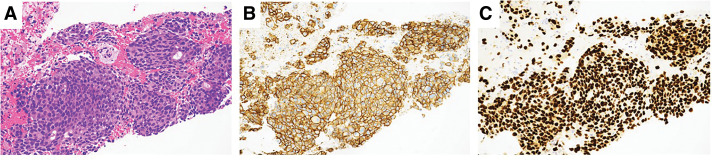
EUS-FNB shows (**A**) pancreatic metastasis from the breast cancer, H&E staining, (**B**) HER2: 3+, and (**C**) GATA3 (magnification, ×20). EUS-FNB, endoscopic ultrasonography-guided fine-needle biopsy; GATA3, GATA-binding protein 3; H&E, hematoxylin and eosin; HER2, human epidermal growth factor receptor 2

Endoscopic ultrasonography revealed a hypoechoic mass with irregular margins in the pancreatic head, a disrupted pancreatic duct, and a dilated caudal main pancreatic duct (**Fig. 4**). Based on the histopathological findings of a fine-needle biopsy (FNB), the pancreatic tumor was a poorly differentiated adenocarcinoma similar to the primary breast cancer: ER-negative; PgR-negative; HER2: 3+; Ki67: 60%; GATA3- and CK7-positive; and CK20-negative, and was diagnosed as pancreatic metastasis from breast cancer (**Fig. 5**). Head MRI also revealed 3 cerebellar metastases (22, 24, and 28 mm in size), which were treated with stereotactic radiation (35 Gy).

**Fig. 6:**
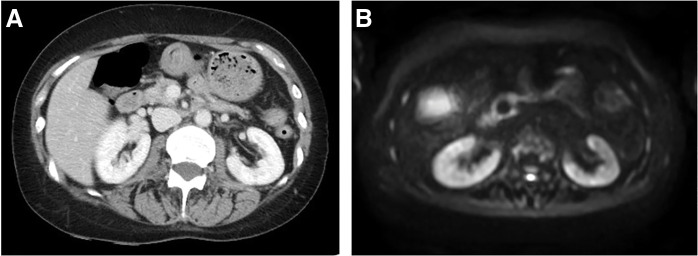
Follow-up CT shows (**A**) a significant reduction in the size of the pancreatic metastasis, and (**B**) MRI reveals that the mass in the pancreatic head has almost disappeared.

T-DXd was administered as 3rd-line treatment. Eight months later, CT revealed a significant reduction in the size of the pancreatic metastasis (**Fig. 6A**). MRI showed that the mass in the pancreatic head had almost disappeared (**Fig. 6B**). Regarding adverse events of the T-DXd, she experienced Grade 1 nausea, Grade 2 malaise, and Grade 1 peripheral neuropathy, but not interstitial pneumonia.

Fourteen months after the administration of T-DXd (41 months after the initial diagnosis), a follow-up CT scan showed that the pancreatic metastasis remained in a CR state, but bilateral pleural effusions had appeared. Pleural fluid cytology revealed pleural dissemination, and capecitabine and lapatinib were administered as 4th-line treatment. Unfortunately, she died of worsening cancerous lymphangitis of the lungs 23 months after the diagnosis of pancreatic metastasis.

## DISCUSSION

We encountered a rare case of pancreatic metastasis from HER2-positive advanced breast cancer that was treated with T-DXd and achieved CR and 14 months of PFS.

From 2008 to 2022, 61 cases of pancreatic metastases in patients with breast cancer have been reported in the literature, including 13 advanced cases and 48 metastatic cases.^10–21)^ Regarding the time of appearance of pancreatic metastases, all patients with advanced breast cancer had pancreatic metastases at the time of the initial diagnosis, whereas pancreatic metastases occurred most frequently at the time of the 1st recurrence (62.5%; 30/48) in patients with MBC.^10,12,13,15,16,18,19,21)^ Of the 30 patients with MBC and pancreatic metastases as the initial recurrence, 12 (25%) also exhibited concurrent recurrence in typical breast cancer metastasis sites, including the bone, liver, LNs, and brain. The remaining patients presented with pancreatic metastases only.

The median time from breast cancer surgery to the diagnosis of pancreatic metastases was 56 months (range: 1–264 months). The median OS after the diagnosis of pancreatic metastases was short, at 17 months (range: 0.3–204 months).

Of the 61 cases of primary breast cancer with pancreatic metastases, 30 were IBC-NSTs,^10–12,16–21)^ as in the present case, while invasive lobular carcinoma^10,12,14)^ accounted for 37.1% (23 cases).

In 45 cases, excluding the 16 cases^10,12,16)^ with no reported subtype, the primary tumor subtypes were ER+HER2− in 24 cases, ER−HER2− in 13 cases, ER−HER2+ in 3 cases, and ER+HER2+ in 5 cases.^10–21)^ Of the 45 patients, 21 underwent FNB or FNA.^10–13,15,16,18,21)^ Sixteen patients underwent FNB, and all were diagnosed with pancreatic metastases from breast cancer.^10–13,16,21)^ Five patients underwent FNA, 3 of whom were diagnosed with primary pancreatic cancer and 2 of whom could not be diagnosed.^10,12,16)^ Twenty-two patients underwent surgical resection of the pancreatic metastases.^10,12,16–19)^ Nine of these patients underwent surgery for differential diagnoses, 8 for misdiagnosis of primary pancreatic cancer, 3 for oligometastases, and 2 for symptomatic relief.^10,12,13,16–19)^ Eighteen patients who did not undergo surgery received systemic therapy for MBC according to their histological subtypes.^10–16,21)^ Four patients underwent palliative therapy.^10,12)^ One patient’s treatment plan was not described in the literature.^12)^ These results suggest that pancreatic metastases are difficult to diagnose.

The present case had HER2 overexpression in both the primary tumor and the pancreatic metastasis. As shown in **Table 1**, in previous reports, there were 7 cases^13,16)^ (5 ER-positive, 2 ER-negative) in which both the primary breast cancer and pancreatic metastases were HER2-positive. In 2 cases of triple-negative breast cancer, the subtype of the pancreatic metastases changed to HER2-positive (1 ER-positive, 1 ER-negative).^10,16)^ In 1 case of HER2-positive breast cancer, the subtype of the pancreatic metastases was not diagnosed because no biopsy or surgery was performed. Five of these 10 patients received anti-HER2 therapy.^12,14–16,21)^ Of these, 1 patient received trastuzumab only, 2 received trastuzumab plus anticancer agents, and 2 received pertuzumab plus trastuzumab plus taxane.^12,13,15,16)^ None of the patients was treated with lapatinib, T-DM1, or T-DXd. The PFS for these 5 cases of anti-HER2 therapy was 5–11 months. In contrast, in the present case, T-DXd resulted in the CR of pancreatic metastasis, achieving a 14-month PFS, which was more effective than that reported in previous cases.

**Table 1:** Patient characteristics in patients with HER2-positive pancreatic metastases

Reference	Sex	Age (years)	DFI (months)	Clinical stage	Histological classification	Subtype of breast cancer	Diagnostic methods for pancreatic metastases	Subtype of pancreatic metastases	1st-line therapy for pancreatic metastases	PFS (months)	OS (months)	Clinical outcome
^5)^	F	51	58	T2N0	IDC	Luminal-HER2	FNB	Luminal-HER2	**Pmab + Tmab + PTX**	6	11	Alive
^7)^	F	60	De novo	T2N1M1	IDC	Luminal-HER2	FNB	Luminal-HER2	**Pmab + Tmab + DTX**	Unknown	Unknown	Unknown
^7)^	F	49	144	T2N0	IDC	Luminal-HER2	surgery	Luminal-HER2	PTX + nedaplatin	14	Unknown	Alive
^8)^	F	62	252	Unknown	IDC	Luminal-HER2	FNB	Luminal-HER2	Chemotherapy (details unknown)	Unknown	68	Death
^11)^	F	40	48	T4N2	IDC	Luminal-HER2	surgery	Luminal-HER2	TAM + LH-RHa	18	Unknown	Alive
^8)^	F	64	De novo	Unknown	IDC	HER2	FNB	HER2	Chemotherapy (details unknown)	72	Unknown	Alive
^11)^	F	53	48	T1bN0	IDC	HER2	FNB	HER2	**Tmab + PTX**	10	24	Death
^5)^	F	51	24	T2N1	IDC	TN	Surgery	Luminal-HER2	UFP + EXE	10	36	Death
^11)^	F	74	72	Unknown	IDC	TN	Surgery	HER2	**Tmab**	5	Unknown	Alive
^10)^	F	61	16	T2N1	IDC	HER2	CT scan (no pathological diagnosis)	Unknown	**Tmab + Cape**	11	Unknown	Alive
Present case	F	52	De novo	T4bN1	IDC	HER2	FNB	HER2	**T-DXd**	14	23	Death

The bold text indicates anti-HER2 therapy drugs.

Cape, capecitabine; DFI, disease-free interval; DTX, docetaxel; EXE, exemestane; F, female; FNB, fine-needle biopsy; HER2, human epidermal growth factor receptor 2; IDC, invasive ductal carcinoma; LH-RHa, luteinizing hormone-releasing hormone agonist; OS, overall survival after diagnosis of pancreatic metastases; PFS, progression-free survival; Pmab, pertuzumab; PTX, paclitaxel; TAM, tamoxifen; T-DXd, trastuzumab deruxtecan; Tmab, trastuzumab; TN, triple negative; UFP, fluorouracil.

The DESTINY-Breast01, 03, and 04 studies demonstrated that T-DXd was effective for visceral metastases, including lung, liver, and brain metastases.^8,9,22)^ In the DESTINY-Breast03 trial, although approximately 70% of patients had visceral metastases, T-DXd showed a higher overall response rate (79.7% vs. 34.2%) and prolonged PFS (12-month PFS: 75.8% vs. 34.1%) compared to T-DM1.^9)^ However, there are no reports on the efficacy of T-DXd for pancreatic metastases in these studies or previous literature. According to the results of the DESTINY-Breast studies and the efficacy of T-DXd in our case, T-DXd may be as effective for pancreatic metastases as for other visceral metastases.

## CONCLUSIONS

Herein, we report a rare case of HER2-positive pancreatic metastasis from breast cancer. T-DXd as a 3rd-line treatment was highly effective in this case, resulting in a long PFS. FNB should be performed to diagnose possible pancreatic metastases if a pancreatic tumor is detected during breast cancer treatment. Further case studies are required to determine the efficacy of T-DXd in the treatment of HER2-positive pancreatic metastases.

## DECLARATIONS

### Availability of data and materials

The dataset supporting the conclusions of this article is included within the article.
